# Supplementary material for: Perceptions of the adult US population regarding the novel coronavirus outbreak
Source: PLoS One. 2020 Apr 17;15(4):e0231808. doi: 10.1371/journal.pone.0231808 (PMC7164638; doi:10.1371/journal.pone.0231808)
Supplement: S1 Survey — (DOCX) [file pone.0231808.s001.docx]

S1 Survey: Perceptions regarding the Novel Coronavirus Outbreak Questionnaire

# Perceptions of the Adult US Population regarding the Novel Coronavirus Outbreak Questionnaire

**Demographics**

**Instructions: Please select the box next to the answer of your choice.**

1. **Gender**

- Male
- Female
- Other

1. **Age**

- 18 – 25
- 26 – 35
- 36 – 45
- 46 – 55
- 55+

1. **Education**

- No high school
- High school
- Some college
- College
- Graduate/Professional

1. **Race**

- Black or African American
- American Indian or Alaska Native
- Asian
- Native Hawaiian or Other Pacific Islander
- White

1. **Ethnicity**

- Hispanic
- Non-Hispanic

**Knowledge of Novel Coronavirus (COVID-19)**

**Instructions: The following are questions that will ask about your knowledge level on novel coronavirus. Please select the answer of your choice.**

1. **Are you aware of the novel coronavirus outbreak?**

- Yes
- No -> *(this explanation pops up if this option is selected)* There is an outbreak of respiratory illness caused by a novel (new) coronavirus first identified in Wuhan, Hubei Province, China. There have been thousands of confirmed cases in China. Additional cases have been identified in other international locations, including the United States.
- Don’t know -> *(this explanation pops up if this option is selected)* There is an outbreak of respiratory illness caused by a novel (new) coronavirus first identified in Wuhan, Hubei Province, China. There have been thousands of confirmed cases in China. Additional cases have been identified in other international locations, including the United States.

1. **How did you learn about the coronavirus outbreak?**

- Media
- Social Media
- Health Officials
- Friends/Neighbors/Relatives

1. **How would you rate your knowledge level on novel coronavirus?**

- Very poor
- Poor
- Average
- Good
- Very good

1. **Which of the following is correct about the definition of novel coronavirus?**

- Novel coronavirus is a respiratory disease caused by a viral infection.
- Displayed symptoms usually include respiratory symptoms accompanied by fever, but novel coronavirus is not contagious.
- Novel coronavirus can progress to a severe illness but never leads to death.
- Don’t know

1. **Which of the following is correct about transmission route of novel coronavirus?**
   - Novel coronavirus is transmitted through coughing or sneezing.
   - Novel coronavirus is not transmitted by close contact with people.
   - Don’t know.

1. **Which of the following is correct about “close contact” of novel coronavirus?**
   - “Close contact” involves a direct contact with persons’ respiratory secretions.
   - Relatives and healthcare workers are excluded from the category of close contact.
   - Don’t know.
2. Which one is correct about the treatment or vaccine for the novel coronavirus?
   - There is a curative treatment for novel coronavirus
   - Currently, there is neither a curative treatment nor a vaccine
   - Currently, there isn’t a curative treatment, but there is a vaccine
   - Don’t know.
3. Which of the following are effective preventative measures for yourself and/or others against the novel coronavirus?

|  | Yes | No | Don’t know |
| --- | --- | --- | --- |
| Hand washing |  |  |  |
| Avoiding touching your eyes, nose, and mouth with unwashed hands |  |  |  |
| Use of disinfectants |  |  |  |
| Staying home when you are sick |  |  |  |
| Herbal supplements |  |  |  |
| Covering your cough |  |  |  |
| A balanced diet |  |  |  |
| Avoiding close contact with someone who is sick |  |  |  |
| Use algae |  |  |  |
| Use caution when opening mail |  |  |  |
| Avoid eating meat |  |  |  |
| Getting the flu shot |  |  |  |
| Regular exercise |  |  |  |
| Wearing a face mask |  |  |  |
| None of the above |  |  |  |

**Risk Perception on Novel Coronavirus**

1. **Instructions: Please select the answer depending on how much you agree with the statements below.**

|  |  | Strongly  Disagree | Disagree | Neutral | Agree | Strongly  Agree | Don’t Know |
| --- | --- | --- | --- | --- | --- | --- | --- |
| 1 | My health will be severely damaged if I contract novel coronavirus. | 1 | 2 | 3 | 4 | 5 |  |
| 4 | I think novel coronavirus is more severe than flu. | 1 | 2 | 3 | 4 | 5 |  |
| 5 | Even if I fall ill with another disease, I will not go to hospital because of risk of getting novel coronavirus in the hospital. | 1 | 2 | 3 | 4 | 5 |  |
| 6 | Novel coronavirus will inflict serious damage in my community. | 1 | 2 | 3 | 4 | 5 |  |
| 7 | Novel coronavirus will spread widely in the United States. | 1 | 2 | 3 | 4 | 5 |  |
| 8 | I am more likely to get the novel coronavirus than other people. | 1 | 2 | 3 | 4 | 5 |  |
| 9 | I believe I can protect myself against the novel coronavirus. | 1 | 2 | 3 | 4 | 5 |  |
| 10 | I believe I can protect myself against the novel coronavirus better than other people. | 1 | 2 | 3 | 4 | 5 |  |

1. **What are some of the measures you have taken to prevent infection from the novel coronavirus? Select all that apply**

|  | Yes | No | Don’t Know |
| --- | --- | --- | --- |
| Avoided travel novel coronavirus infected areas. | Yes | No | Don’t Know |
| Washed hands with soap and water. | Yes | No | Don’t Know |
| Used disinfectants. | Yes | No | Don’t Know |
| Avoided touching your eyes, nose, and mouth with unwashed hands. | Yes | No | Don’t Know |
| Avoided eating outside of the home. | Yes | No | Don’t Know |
| Stayed home when you were sick. | Yes | No | Don’t Know |
| Covered your cough or sneeze with a tissue, then throw the tissue in the trash. | Yes | No | Don’t Know |
| Avoided close contact with people who are sick. | Yes | No | Don’t Know |
| Took an herbal supplement. | Yes | No | Don’t Know |
| Exercised regularly. | Yes | No | Don’t Know |
| Ate a balanced diet.  Used algae.  Used caution when opening mail.  Avoided eating meat.  Wore a face mask | Yes | No | Don’t Know |
| None of the above. | Yes | No | Don’t Know |

1. **Have you been vaccinated against the flu virus in the last 6 months?**

- Yes
- No
- Don’t know

1. **Do you plan to get a flu shot in the next 4 months?** *(pops up only if “no” or “don’t know” is selected for previous question)*

- Yes
- No
- Don’t know

**Sources of Information**

**Instructions : Please select the answer depending on how much you agree with the statements below.**

1. For the following sources of information in United States, please rate how reliable you feel they are with respect to the novel coronavirus.

|  | Very Little | Little | Some | Much | Very Much | Don’t know |
| --- | --- | --- | --- | --- | --- | --- |
| 1) Television |  |  |  |  |  |  |
| 2) Newspapers/Magazines |  |  |  |  |  |  |
| 3) Websites |  |  |  |  |  |  |
| 4) Friends/Family |  |  |  |  |  |  |
| 5) Health care professionals |  |  |  |  |  |  |
| 6) Health Officials (government, Centers for Disease Control and Prevention) |  |  |  |  |  |  |
| 7) Social Media |  |  |  |  |  |  |

**Trust**

1. How much confidence do you have in each of these organizations?

|  | Very Little | Little | Some | Much | Very Much | Don’t know |
| --- | --- | --- | --- | --- | --- | --- |
| Your own doctor |  |  |  |  |  |  |
| Local/County health department |  |  |  |  |  |  |
| State health department |  |  |  |  |  |  |
| Centers for Disease Control and Prevention (CDC) |  |  |  |  |  |  |
| Food and Drug Administration (FDA) |  |  |  |  |  |  |
| The White House |  |  |  |  |  |  |
| Department of Health and Human Services |  |  |  |  |  |  |
| National Institute of Health |  |  |  |  |  |  |
| Professional Organizations (example: American Medical Association or the Infectious Disease Society of America) |  |  |  |  |  |  |

1. Please rank each of these organizations in order of your confidence in them.

|  | Rank |
| --- | --- |
| Your own doctor |  |
| Local/County health department |  |
| State health department |  |
| Centers for Disease Control and Prevention (CDC) |  |
| Food and Drug Administration (FDA) |  |
| White House |  |
| Department of Health and Human Services |  |
| National Institute of Health |  |
| Professional Organizations (example: American Medical Association or the Infectious Disease Society of America) |  |

**Outrage**

1. **Instructions: Please select the answer depending on how much you agree with the statements below.**

|  | Strongly  Disagree | Disagree | Neutral | Agree | Strongly  Agree | Don’t Know |
| --- | --- | --- | --- | --- | --- | --- |
| I am very concerned about this outbreak. | 1 | 2 | 3 | 4 | 5 |  |
| I believe that the government is exaggerating the threat. | 1 | 2 | 3 | 4 | 5 |  |
| I trust that health officials can effectively handle this crisis. | 1 | 2 | 3 | 4 | 5 |  |
| I trust the Congress to effectively handle this crisis. | 1 | 2 | 3 | 4 | 5 |  |
| I trust that the President can effectively handle this crisis. | 1 | 2 | 3 | 4 | 5 |  |
| I expect this outbreak to get larger. | 1 | 2 | 3 | 4 | 5 |  |
| I agree that United States should travel from China. | 1 | 2 | 3 | 4 | 5 |  |
| Americans who visited China during this outbreak should be quarantined. | 1 | 2 | 3 | 4 | 5 |  |
| In case of an outbreak, it is reasonable to temporarily discriminate against a community based on their country of origin | 1 | 2 | 3 | 4 | 5 |  |

1. From your perspective, rank in order of who you think should overall be in charge of America’s outbreak response.

|  | Rank |
| --- | --- |
| The President |  |
| The Secretary of Health and Human Services |  |
| Director of National Institute of Health (NIH)/National Institute for Allergy and Infectious Diseases |  |
| Director of the Centers for Disease Control and Prevention (CDC) |  |
| State Health Departments |  |
| Local Health Departments |  |
| Other |  |
| Don’t know |  |

Thank you for participating in this survey.

**Survey Citation**: McFadden, S. M., Malik, A. A., Aguolu, O. G., Willebrand, K. S., & Omer, S. B. (2020). Perceptions of the Adult US Population regarding the Novel Coronavirus Outbreak Questionnaire.
