# Supplementary material for: Perceptions of the adult US population regarding the novel coronavirus outbreak
Source: PLoS One. 2020 Apr 17;15(4):e0231808. doi: 10.1371/journal.pone.0231808 (PMC7164638; doi:10.1371/journal.pone.0231808)
Supplement: Attachment — Submitted filename: COVID-19 reponse to reviewers.docx [file pone.0231808.s004.docx]

March 25, 2020

Dear Dr. Samy,

We would like to thank you and the reviewer for your careful review of this manuscript and the insightful comments to help improve our work. We have attempted to address all the concerns that have been raised. We believe that the manuscript is stronger as a result. Please see our point-by-point responses below.

On behalf of the authorship team,

Saad Omer, MBBS, MPH, PhD, FIDSA
Director | Yale Institute for Global Health
Associate Dean (Global Health Research) | Yale School of Medicine

Professor of Medicine (Infectious Diseases) | Yale School of Medicine

Adjunct Professor | Yale School of Nursing

Susan Dwight Bliss Professor of Epidemiology of Microbial Diseases | Yale School of Public Health

**Responses to Reviewer’s Comments**

Reviewer 1:

1. In abstract line 1: please add Coronavirus Disease 2019 as a definition for COVID-19.

Response: The sentence has been revised as requested. The sentence now reads as follows:

*“The Coronavirus Disease 2019 (COVID-19) outbreak is spreading globally.”* (line 20)

2. In abstract line 2: the risk of infection in US, I think now changed to high due to pandemic declaration (also amend in all manuscript).

Response: We amended this both in the abstract and manuscript, but we did clarify that at the time of survey administration the risk for contracting COVID-19 was thought to be low. The sentence now reads as follows:

*“Although COVID-19 has now been declared a pandemic and risk for infection in the United States (US) is currently high, at the time of survey administration the risk of infection in the US was low.”* (lines 20 – 22)

3. In Background page 3, line 1: the countries infected by COVID-19 need update according to the latest WHO report (not 28 countries).

Response: We amended this by stating it has spread across the globe and updated the reference. The sentence now reads as follows:

*“The current novel coronavirus outbreak, COVID-19, has spread across the globe with hundreds of thousands infected and thousands of deaths (1).”* (lines 39 – 40)

4. In Background paragraph 1: please add a sentence contain the actual numbers of infections and mortality rates globally and in US with recent reference.

Response: We added this sentence for the US with the most recent reference from the CDC. It now reads as follows:

*“At the time of this writing, there are over 54,000 cases of COVID-19 in the US with 737 deaths (2).With COVID-19 now declared a pandemic by the World Health Organization (3), it is important to understand risk perceptions about COVID-19 and trust in political and public health/scientific leadership among the US population to better inform messaging and policies (4).”* (Lines 40 – 44)

5. In page 5 and 6, Figure 1, 2: there are no error bars and the statistically significant marks, also describe the statistical parameters used in analysis (type parameters in Legends).

Response: We have added the error bars to figures 1,2 and 4 and revised figure 3 so that all error bars now show SE and are consistent. As the objective of this study was to provide descriptive data on the perceptions of the US population, we did not carry out any inferential statistical testing and hence do not have a statistically significant marks on the figures to report.
